# Toll-like receptors in lupus nephritis

**DOI:** 10.1186/s12929-018-0436-2

**Published:** 2018-04-12

**Authors:** Satish Kumar Devarapu, Hans-Joachim Anders

**Affiliations:** Nephrologisches Zentrum, Medizinische Klinik und Poliklinik IV, Klinikum der Ludwig Maximilians Universität München, Ziemssenstr, 1&Schillerstr, 42, 80336 Munich, Germany

**Keywords:** Immune complex, Inflammasome, Innate immunity, Interferon, Glomerulonephritis

## Abstract

The pathogenesis of systemic autoimmune diseases such as systemic lupus erythematosus (SLE) is based on the loss of self-tolerance against ubiquitous autoantigens involving all mechanisms of adaptive immunity. However, data accumulating over the last decade imply an important role also for numerous elements of innate immunity, namely the Toll-like receptors in the pathogenesis of SLE. Here we discuss their role in the most common organ complication of SLE, i.e. lupus nephritis. We summarize experimental and clinical data on the expression and functional contribution of the Toll-like receptors in immune complex glomerulonephritis, and intrarenal inflammation. Based on these discoveries Toll-like receptors are evolving as therapeutic targets for the treatment of SLE and lupus nephritis.

## Background

Toll-like receptors (TLR) have an essential role in innate immunity during host defense which impacts also on auto-inflammatory and autoimmune diseases [1]. Once *Tlr-*deficient mice became available the hypothesis of TLRs as boosters of systemic autoimmunity [2] including disease such as systemic lupus erythematosus (SLE) became possible. In the first part of this review, we discuss the role of TLRs in SLE and in the second part their contribution to intrarenal inflammation in lupus nephritis (LN). Further, we have also briefly mentioned about TLR role in immune tolerance break and germinal center formation in the second part.

### Toll-like receptors in systemic autoimmunity upstream to nephritis

**Fig. 1:**
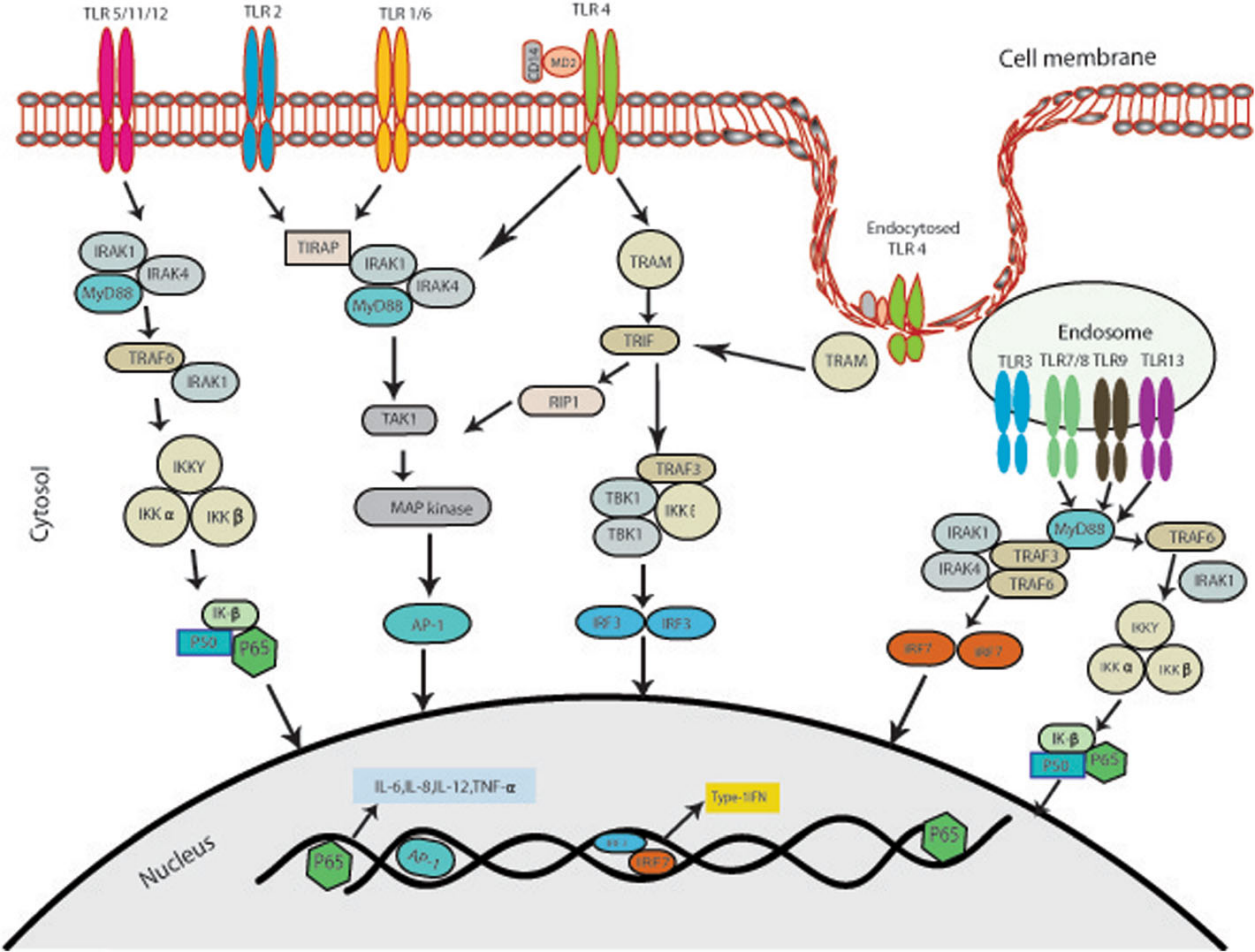
Toll-like receptor (TLR) signalling pathways. TLR1, TLR2, TLR4, TLR5, TLR6, TLR11, TLR12 as homo or heterodimers bind to their respective ligands at the cell surface, whereas TLR3, TLR7/TLR8, TLR9, and TLR13 sense nucleic acids in endosomes. TLR4 localizes at both the plasma membrane and the endosomes. The Toll–IL-1-resistence (TIR) domains of TLRs-dimers bind TIR domain-containing adaptor proteins (either myeloid differentiation primary-response protein 88 (MYD88), or TIR domain-containing adaptor protein inducing IFNβ (TRIF) and TRIF-related adaptor molecule (TRAM)). Downstream signalling pathways of TLRs involve IL-1R-associated kinases (IRAKs), IκB kinase -alpha, beta, gamma and epsilon (IKK αβγε-) and the adaptor molecules TNF receptor-associated factors (TRAFs). These protein complexes activate p50 and p65, as well as various transcription factors such as interferon-regulatory factors (IRFs) and activator protein 1 (AP1). TLR signalling leads to the induction of pro-inflammatory cytokines or the induction of type I interferon (IFN)

Based on the location of expression, TLRs are classified into extra-cellular and intra-cellular (Fig. 1).

### Extracellular TLRs

#### Toll-like receptors 2 and 4

TLR-2 and TLR-4 are membrane-bound receptors that recognize bacterial wall components and endogenous components of dying cells, so-called damage-associated molecular patterns (DAMPs) [3, 4]. In C57BL6^lpr/lpr^ mice, a model of spontaneous autoimmunity, TLR2 or TLR4 deficiency limits the expansion of the marginal zone B cell compartment and attenuates the levels of antinuclear-antibodies and rheumatoid factors [5]. However, it should be noted that C57BL6^lpr/lpr^ mice are a very mild strain in developing the disease compared with MRL-lpr strain. Interestingly, anti-nucleosome antibodies are diminished in the absence of TLR2 but not of TLR4 [5]. Administration of pristine a natural saturated terpenoid alkane to genetically intact mice results in the development of hypergammaglobulinaemia with the production of lupus-like autoantibodies and proliferative glomerulonephritis, with similarities to human lupus nephritis. However, administration of the pristine in *Tlr4*- deficient mice demonstrated a global decrease in both Th1, IFNγ, and Th17 associated IL-17A and IL-6 cytokine production. Also, autoantibody levels of anti-dsDNA and anti-RNP were both decreased indicating the requirement of TLR4 for the full-blown autoimmunity [6]. Furthermore, exposing anti-ds DNA transgenic mice to TLR4 agonistic LPS aggravates systemic autoimmunity and LN [7].

Using transgenic mouse called TCr-5, whose genome contains multiple copies of the tlr4 gene on a TLR4-deficient C57/BL10ScCr (deletion of tlr4 gene and point mutation in IL-12Rβ 2gene) background, it has been shown that up-regulation TLR4 is pathogenic both at the protein level and gene level and triggered spontaneous SLE-like autoimmunity and lupus nephritis [8]. Mice deficient in Lyn, a serin threonine kinase with an inhibitory function on TLR4/IL-1 signaling in dendritic cells, develop spontaneous lymphoproliferation, nephritis and autoantibody production. Indeed, the effects of Lyn-deficiency could be restored by simultaneous deletion of MyD88 in dendritic cells [9]. Obviously, the immunostimulatory functions of TLR4 can trigger the initiation of autoimmunity in permissive genetic constellations [10] even when aberrant TLR-4/IL-1 signaling is restricted only to dendritic cells [9]. However, *Tlr2* or − *4*-deficiency in MRL/lpr mice did not significantly affect disease severity [11].

In human overt SLE, TLR2 mRNA is upregulated in PBMCs [12] but in young lupus patients (median age of 13 years at the time of enrolment) monocyte TLR2 and-4 cell surface expression can be even reduced when examined by flow cytometry [13]. Stimulation of PBMCs from SLE patients with TLR2 or − 4 agonists triggers more cytokine production as compared to healthy subjects [14]. TLR2 is also increased on CD4+ and CD8+ positive T cells and B cells from SLE patients, which is associated with the increased in-vitro production of IL-17 upon activation with TLR-2 agonists [15]. South Indians bearing the TLR4 promoter polymorphisms D299G and T399I show an increased risk for specific autoantibody production (Table 1) [16]. Together, accumulating data suggest an involvement of TLR2 and − 4 in the pathogenesis of systemic autoimmunity in mice and humans.

**Table 1:** Various TLR polymorphisms studied in SLE and LN patients

TLR	Allele	Ethnicity/year of study	Association
TLR2	rs1816702,rs4235232	multiethnic group,2014	positive [149]
	R753Q	South Indians,2015	positive [150]
TLR3	rs3775296-T	Taiwanese,2014	positive [26]
	rs3775291	Danish,2014	positive [151]
	rs3775291	Meta analysis, 2016	No association [60]
TLR4	D299G,T399I	Meta analysis 2016	positive [152]
	rs4986790,rs4986791	Meta analysis, 2016	No association [60]
	D299G,T399I	South Indian cohort,2015	positive [16]
	1196 C/T	Polish population,2013	Negative [153]
TLR5	rs5744168	American^a^,2017	No association [19]
	rs5744168	Zhuang and Han ethnics,2015	No association [154]
TLR7	PTPN22 Variant R620W	American^a^,2015	positive [155]
	rs3853839	Danish,2014	positive [156]
	s3853839-G,rs3853839,rs179010-T	Taiwanese,2014	positive [26]
	rs3853839	Mutliethnic group,2013	positive [157]
TLR8	rs3764880-G,rs3764880	Taiwanese,2014	positive [26]
	rs3764879	Danish,2014	positive [156]
	rs37648	Danish,2014	positive [151]
TLR9	rs352143	Danish,2014	positive [151]
	rs352140	American^a^,2017	positive [19]
	rs352140	Zhuang and Han ethnics,2015	positive [154]
	PTPN22 rs2476601	Meta analysis,2017	positive [158]
	rs187084	South Indians,2017	positive [159]
	rs352139	Egyptian,2016	positive [160]
	-1237C/T	South Indians,2015	positive [150]
	T-1237C and T-1486C	Meta analysis,2013	negative [161]
	rs352140, rs5743836,rs352139	Meta analysis, 2016	negative [60]

^a^ethnicity was not clear; study information was restricted for the last five years

#### Toll-like receptor-5

Little is known about TLR5 in lupus but some contribution to SLE seems possible. Renal TLR5 expression is upregulated in nephritic MRL/lpr mice, probably because of immune cell infiltrates. In vitro treatment of murine macrophages with the TLR5 ligand flagellin induces IL-6 production [17] indicating that microbial components can efficiently ligate with activated immune cells present in immune complex glomerulonephritis. In humans, TLR5 maps to a q41-q42 locus on chromosome 1 that includes strong SLE susceptibility genes. Transmission disequilibrium testing in a cohort of 199 SLE patients versus 75 healthy siblings implies a protective role for SNP1174T in the TLR5 gene, probably because SNP1174T transmits a stop codon abrogating TLR5 signaling [18]. The rs5744168 is most studied SNP for TLR5 and found no genotype-phenotype association in SLE however, the T allele and the TT genotype were raised significantly in LN patients group indicating the putative role of TLR5 in the progression of LN [19]. Even though the available data on TLR5 with respect to SLE are limited the existing data suggest a contribution of TLR5 to SLE manifestations.

### Intracellular TLRs

#### Toll-like receptor-3

TLR3 receptor localizes to intracellular endosomes and senses double-stranded RNA via the adaptor TRIF instead of MyD88 [20]. This way TLR3 employs the transcription factor IRF3 to induce type I interferon secretion, a central element in the pathogenesis of SLE [10]. Although single vaccination-like doses of the TLR3 ligand polyI:C RNA do not exacerbate autoimmunity in MRL/lpr or FcγR2b−/− mice with SLE-like autoimmunity, repetitive doses of polyI:C RNA do aggravate kidney disease in MRL/lpr mice but independently from B cell-driven immune complex disease [21, 22]. It, therefore, was concluded that viral dsRNA triggers disease activity of lupus nephritis by mechanisms that are different from those of bacterial DNA. The gene product of the ITGAM gene, CD11b, is an endogenous negative regulator of TLR3 signaling and gene variants in the ITGAM gene affect the risk for SLE in humans [23] suggesting activation of CD11b could be a potential mechanism for developing SLE therapeutics. In SLE patients also TLR3 expression is upregulated in total PBMCs, T- and B cells [24, 25]. The TLR3 gene rs3775296-T risk allele was found to be associated with anaemia and photosensitivity in Taiwanese SLE patients [26] indicating that TLR3 involved the development of different SLE phenotypes, and in antiviral responses that trigger expression of pro-inflammatory genes. Taken together these data suggest that viral ds RNA trigger disease activity through TLR3 in a MyD88 independent pathway involving various innate immune cells.

#### Toll-like receptor-7

TLR7 locates together with TLR3 (and TLR9) inside intracellular endosomes. TLR7 senses viral single-stranded RNAs and triggers cytokine and type I interferon production for antiviral host defense but endogenous nucleic acids can have the same effect [27, 28]. TLR7 activation with imiquimod activates systemic type I interferon production and aggravates lupus nephritis in MRL/lpr mice [29]. Monoclonal antibodies targeting TLR7 get internalized after binding to a surface-bound TLR7 fraction [30]. TLR7 activity mediates the formation of anti-RNA autoantibodies and glomerulonephritis in MRL/lpr mice as well as in pristane-induced SLE [31, 32]. MRL/lpr mice deficient for TLR7- and TLR9-signalling do not develop antinuclear antibodies [33]. TLR9 activity inhibits TLR7-mediated autoimmunity [33]. Therefore, MRL/lpr mice lacking TLR9 develop increased autoantibody levels directed against RNA autoantigens such as anti-Sm or anti-ribosomal P Ag [33].

TLR7 overexpression in B6.yaa mice illustrates that increased TLR7 activity alone is not sufficient to trigger autoimmunity without gene variants in other susceptibility genes [34]. The y-linked autoimmune accelerator (yaa) allele is the most potent disease allele on the Y chromosome detected in BXSB mouse strain involved in the pathogenesis of lupus nephritis; however, yaa could produce disease only when combined with other autoimmune promoting genes [35, 36].TLR7-driven type I interferon secretion can also promote hematopoietic abnormalities [37]. TLR3/TLR7 co-activation has synergistic immunostimulatory effects in MRL/lpr mice [38].

TLR7 is expressed in several B cell subtypes, macrophages, and plasmacytoid dendritic cells [39]. In MRL/lpr mice dendritic cells promote expansion and differentiation of T cells, but not their activation [40]. Interestingly, in MRL/lpr mice TLR/MyD88 signaling in dendritic cells is dispensable for the development of autoantibody production and lupus nephritis [41]. TREML4 augments TLR7 signaling during autoimmune disease. Indeed, *Treml4*-deficient MRL/lpr mice have reduced T cell numbers, lower systemic IFN-α levels, show less nephritis [42]. Mice, lacking the negative regulator Lyn, spontaneously develop autoimmunity [43] and conditional deletion of MyD88 in dendritic cells in *Lyn*-deficient mice decreases germinal center formation, DNA autoantibody production, and immune complex glomerulonephritis [44]. A similar phenotype is present in mice deficient for the negative regulator interleukin-1 receptor-associated kinase-M [45] and single Ig IL-1-related receptor [46]. In addition, abolishing the function of plasmacytoid dendritic cells in TLR7.Tg mice, a model of TLR7-driven autoimmunity, attenuated glomerular immune complex deposits and prolonged lifespan of the mice [47].

In contrast, genetic depletion of MyD88 in B cells impairs the secretion of antinuclear antibodies and abrogates lupus nephritis because activated B cells contribute to both antibody-dependent and -independent renal T cell infiltrates in MRL/lpr mice [41]. Indeed, in *Tlr7* transgenic mice autophagy in B cells is an initiating event triggering TLR7-dependent autoantibody production [48]. B cell receptor-driven uptake of immune complexes stimulates TLR7 and − 9 inside B cells and drives RNA- and DNA-autoantibody production, respectively [33, 49–54]. In this context, germinal center formation in health and disease involves B cell TLR7 signaling [55]. Thus, normalizing CD19+ B cell TLR7 expression in transgenic *Tg7Sle1B6* mice overexpressing TLR7 in a lupus-prone background normalized lymphocyte activation but not proliferation. Nevertheless, less production of RNA autoantibodies somewhat reduced the activity of immune complex glomerulonephritis [56].

PBMCs from different ethnic SLE populations consistently overexpress TLR7 along with a type I interferon transcript signature [57, 58]. TLR7 gene variants can be risk factors for human SLE [26, 59]. A recent meta-analysis comprising 11,984 patients documented a link between the TLR7 variants rs179008 and the risk of SLE in African populations and between rs3853839 and SLE in Asians [60]. Japanese male SLE patients carrying a single nucleotide polymorphism in the 3’UTR of the TLR7 gene corresponds to an increased TLR7 gene dose and type-I interferon gene signature [61]. Furthermore, a Mexican case-control study of 328 childhood-onset SLE patients and 403 controls displayed an increase in TLR7 copy numbers as an SLE risk factor [62]. It is of note that, another study could not confirm these in adult-onset SLE [63]. Interestingly, the gene product of the ITGAM gene, CD11b, is an endogenous negative regulator of TLR7 signaling and gene variants in the ITGAM gene affect the risk for SLE in humans [23]. Intrinsic IgE seems to be another endogenous inhibitor of TLR7-mediated IFN-α induction in dendritic cells isolated from SLE patients [64]. In summary, TLR7 is involved in the pathogenesis of murine and human SLE and LN by sensing endogenous ribonucleoprotein antigens for the activation of antigen presenting cells such as B cells and dendritic cells [10, 65, 66]. In this process, the nucleic acid component acts as an endogenous adjuvant to boost immunity against ribonucleoproteins [67]. The phenomenon of TLR7 sensing danger signals of RNA autoantigens is similar to sensing signals delivered during viral infection and could therefore be referred to as molecular mimicry in innate immunity.

#### Toll-like receptor-8

In humans, TLR8 senses ssRNA [68–70] but its role in autoimmunity is less clear. 564Igi mice, a knock-in strain expressing an autoreactive anti-RNA-antibody, require TLR8 and MyD88 to break tolerance and to induce a type-I interferon signature [71]. Autoantibody secretion is abrogated upon simultaneous deletion of TLR7 and TLR8 but not of TLR7 and TLR9 or other combinations [71]. However, TLR8 deletion of C57BL/6 genetic background triggers anti-dsDNA antibody secretion and glomerulonephritis, an effect abrogated by co-deletion of TLR7 suggesting rather an immunoregulatory role of TLR8 in TLR7 [72]. Indeed, dendritic cells isolated from *Tlr8*−/− mice present increased TLR7 expression and are hyper-responsive to TLR7 agonists [72]. In contrast to TLR9, which mainly executes its regulatory effect on TLR7 in B cells, TLR8 inhibits TLR7 mostly in dendritic cells [10, 73]. Evidently, TLR9 and TLR8 inhibit TLR7-mediated autoimmunity and renal inflammation in a synergistic manner [71, 73].

TLR8 is also present in humans [26, 74, 75] and TLR8 expression levels are upregulated in blood cells of paediatric and adult lupus patients [76]. Females express higher levels of TLR8 mRNA in blood cells in the presence of 17β-estradiol as a potential factor in gender disparity of SLE [74]. In addition, the TLR8 single nucleotide polymorphism rs3764880 is associated with an increased risk for SLE in females but not in males [26]. As another aspect, certain antiphospholipid antibodies upregulate miR-146a-3p in trophoblast cells, which enhances TLR8-mediated IL-8-secretion [75]. Together, also TLR8 seems to have an immunoregulatory role in systemic autoimmunity, especially in females.

#### Toll-like receptor-9

AlsoTLR9 localizes to the endosomal compartment where it senses unmethylated CpG motifs in the DNA sequence of viruses or bacteria [10]. However, endogenous TLR9 ligands exist, e.g. hypomethylated self-DNA within immune complexes, neutrophil extracellular traps, oxidized mitochondrial nucleoids or other chromatin formats [10, 28, 77–79]. Defects in lysosomal maturation endorse the activation of TLR9 [80]. TLR9 ligation is followed by type I interferon production in immune cells especially plasmacytoid dendritic cells [81]. Indeed, TLR9 and TLR7 in SLE partially mimic a state of anti-viral host defense referred to as “pseudoantiviral immunity” [82, 83]. Type I interferons promote the responsiveness to B-cell receptor crosslinking in B cells and render dendritic cells responsive to endogenous nucleic acids after upregulation of TLR7 and TLR9 [84, 85]. TLR9-dependent type I interferon production depends on a lipid kinase - PIKfyve, a mechanism that can neutralized by *apilimod* [86]. However, not all features of human SLE are attributable to type I Interferons as longitudinal studies do not always show I interferon signatures to indicate disease activity in patients [87, 88]. Blocking endogenous TLR9 ligands with a TLR9 antagonist in MRL/lpr mice ameliorated systemic autoimmunity and immune complex glomerulonephritis [89]. Consistently, injections with CpG and LPS to anti-dsDNA transgenic mice exacerbated disease [7]. In line, *Tlr9*-deficient C57BL/6 mice display less autoimmunity and nephritis due to less glomerular Ig-deposits and complement activation in pristane-induced autoimmunity [6]. However, BALB/c mice injected with pristane show different effects, highlighting the contribution of additional genetic factors [90]. Nevertheless, dual inhibition of both TLR7 and TLR9 did not elicit additive effects beyond single blockade of TLR7 in MRL/lpr mice. TLR9 inhibition even neutralized the beneficial effects of the TLR7 blockade on systemic autoantibody levels without affecting the effects of the TLR7 blockade on nephritis [91]. Hence, the regulatory role of TLR9 is context-dependent.

It is of note that TLR9 deletion in MRL/lpr mice aggravates systemic autoimmunity and immune complex glomerulonephritis, a phenomenon that can be neutralized co-deletion of TLR7 [33]. Obviously, TLR9 and TLR7 normally compete for endosomal trafficking implying that increased TLR7 signaling driving autoimmunity can outweigh the pro-inflammatory effects of TLR9 [92, 93]. In addition, *Tlr9*- and *Tlr8*-double-deficient C57BL/6 mice develop spontaneous signs of autoimmunity such as splenomegaly, autoantibody production, and glomerulonephritis. Here, TLR9 seems to restrict B cell TLR7 signaling, while TLR8 suppresses TLR7-driven activation of dendritic cells [73]. However, B cell TLR9 is required for the generation of DNA antibodies in mice and promotes the differentiation of autoantibody-secreting B and plasma cells in humans [94, 95]. TLR9 mRNA expression is also upregulated in PBMCs from SLE patients, correlates with lupus nephritis [57, 96], and correlates with circulating anti-DNA autoantibody levels [96]. B cell TLR9 expression is associated with serum creatinine levels in SLE patients [25]. Intrinsic IgE inhibits also TLR9-mediated IFN-α induction in dendritic cells isolated from SLE patients [64]. Together, TLR has a complex role in SLE as it promotes DNA autoantibody production as an important component of lupus nephritis. However, TLR9 also inhibits TLR7 signaling as another central pathway in the pathogenesis of SLE. Therefore, therapeutic TLR9 inhibition may cause unexpected effects in human SLE depending on the predominant role of these different elements of disease pathogenesis.

### Toll-like receptors inside the nephritic kidney

How do TLRs maintain tissue inflammation inside the kidney once lupus nephritis has established? Extrinsic and intrinsic ligands can ligate TLRs on infiltrating monocytes, dendritic cells and B cells to enhance cytokine secretion [17, 97, 98]. Furthermore, mesangial cells and other parenchymal cells express TLR1–4, and TLR6 [17]. In culture, renal cells respond to TLR activation and secrete interleukins and chemokines [17, 99]. Also, immune complex deposits containing TLR agonists activate glomerular mesangial cells and contribute to immune complex glomerulonephritis [100, 101].

Inside the kidney, TLR2 and TLR4 are expressed not only by parenchymal cells but also by infiltrating neutrophils and mononuclear phagocytes including macrophages and dendritic cells [10, 102]. Murine macrophages respond to TLR2 and − 4 agonists by secretion of pro-inflammatory cytokines [17]. Also, HMGB1, a DNA-binding protein, and lupus autoantigen, released under inflammatory conditions can induce NF-κB activation in a TLR2 and TLR4-RAGE-dependent manner in mononuclear phagocytes and neutrophils [103, 104] as well as in mesangial cells [103, 105]. When HMGB1 combines with the nephritogenic anti-DNA antibody 1A3F they form complexes that activate the TLR2 axis in mesangial cells via the FoxO1 signaling pathway [83, 105, 106]. Mesangial cells and podocytes express also TLR4 in the nephritic environment [17, 107]. Mesangial cells isolated from NZB/W mice with spontaneous autoimmunity express significantly higher levels of TLR4 and produce significantly more pro-inflammatory chemokines under baseline and LPS-stimulated conditions [108]. Also, necrotic cell debris is enriched with endogenous TLR ligands that stimulate cytokine release via TLR2/MyD88 depend on the way in mesangial cells, a finding that probably implies also to other intrarenal cell types expressing TLR2 [109]. Intrarenal TLR2 and TLR4 expression are also enhanced in kidneys from FcgammaRIIb-deficient mice suffering from cryoglobulinemic glomerulonephritis [107]. In MRL/lpr mice bacterial lipopeptide aggravates albuminuria in nephritic animals via TLR2-mediated activation of podocytes and endothelial cells at the glomerular filtration barrier [110]. These data help to understand how TLR2 and − 4 on infiltrating immune cells and parenchymal cells amplify local inflammation and barrier dysfunction by sensing extrinsic and intrinsic danger signals serving as TLR agonists.

An inflammatory microenvironment stimulates TLR3 expression in murine and human mesangial cells [17, 111]. Repetitive injections with the TLR3 agonist poly I:C RNA aggravates glomerulonephritis in MRL/lpr mice without affecting systemic autoantibody levels, hence, circulating viral double-stranded promote lupus nephritis mainly via local cytokine production by infiltrating macrophages and resident renal mesangial cells [22]. It is of note that mesangial cells from female animals express more TLR3 copies and secrete more IL-6 production upon TLR3 activation, providing another explanation for gender disparity in lupus. Indeed, deficiency of estrogen receptor-α in mesangial cells abrogates TLR3 signaling in mesangial cells [112]. Also, chloroquine attenuates TLR3-mediated type I interferon induction in human mesangial cells [113, 114]. TLR3 ligation in human mesangial cells promotes the secretion of the neutrophil chemoattractant CXCL1 and CXCL1 is also expressed in diagnostic biopsies from patients with lupus nephritis but not in biopsies of patients with IgA nephropathy [99]. These data support the concept of pseudo-antiviral immunity also at the tissue level because type I interferons from resident renal cells drive renal damage in experimental antibody-triggered glomerulonephritis [98, 114, 115]. Tubuloreticular inclusions represent a morphological imprint of local type I interferon activity and can be found only in lupus nephritis, viral nephropathies, and upon IFN-α treatment [116]. It is of note that type I interferon production in mesangial cells does not require TRIF or MyD88 when it involves non-TLR cytosolic RNA recognition receptors [117, 118].

In murine and human lupus nephritis plasmacytoid dendritic cells type recognize ssRNA, CpG DNA from bacteria and viruses as well as altered eucaryotic nucleic acids via TLR7 and − 9 [119], which induces the release of type I interferons and foster local and systemic immune responses via enhanced expression of costimulatory molecules [116, 119]. Specific deletion of plasmacytoid dendritic cells in a murine TLR7-driven lupus model (Tlr7.Tg) or a polygenic model (B6.*Sle1.Sle3*) attenuates not only systemic autoimmunity but also lupus nephritis [47]. Transient depletion of plasmacytoid dendritic cells can somewhat suppress glomerulonephritis [120]. Also, conventional dendritic cells express TLR7 in *Sle1*Tg7 mice. TLR7 overexpression is associated with an accumulation of CD11b + conventional dendritic cells inside the kidneys. Correcting the abnormal expression levels of TLR7 selectively in CD11c + conventional dendritic cells protected kidneys from autoimmune inflammation [39]. Thus, TLR7-mediated RNA sensing in conventional dendritic cells is an essential component in lupus nephritis in mice [39]. Similar studies were conducted to evaluate the alterations in circulating or possibly tissue infiltrating DCs in human lupus nephritis patients and found that conventional and plasmacytoid dendritic cells decreased in circulation enriched in both, the glomerular and tubulointerstitial, compartments in diagnostic kidney biopsies of SLE patients with lupus nephritis suggesting locally recruited DCs might play a pathogenic role in LN [121].

Circulating nucleic acids inside immune complexes can ligate TLR9 inside antigen-presenting cells (mononuclear phagocytes and B cells) to trigger pro-inflammatory cytokines as well as antibody secretion [10, 17]. TLR9 is absent in mesangial cells of MRL/lpr mice but other groups found TLR9 in tubular epithelial cells of NZBxNZW mice and in diagnostic biopsies of patients with lupus nephritis [17, 122–124]. In childhood-onset lupus nephritis injured podocytes stained positive for TLR9, while TLR9 was absent in podocytes of healthy control kidneys or during remission of lupus nephritis [125, 126]. BXSB-Yaa mice with SLE express TLR8 mRNA and protein in podocytes and TLR8 expression was found to correlate with podocyte injury and negatively correlate with albuminuria [127]. Overall, TLR7, − 8, and 9 drive lupus nephritis because endogenous (and during infections possibly exogenous) nucleic acid-based TLR agonists activate infiltrating antigen-presenting cells inside the nephritic kidney.

### TLRs in immune tolerance break and germinal center formation

Other aspects of TLR involvement in autoimmune diseases are immune tolerance and germinal centers formation. Here we would like to provide readers a brief summary of these two aspects. Failure or loss of central and peripheral immune system results in the uncontrolled activation of self-reactive B and T cells and hence results in autoimmunity. Different TLRs have been shown to involve in this breakdown of tolerance [128–130]. For example, repeated stimulation of TLRs induces unresponsiveness to the same TLR ligand in cell lines [131, 132], B cells [133] and plasmacytoid DCs [134, 135].In some observations, it was found that repeated stimulation of a specific TLR can even result in cross-tolerance but also in enhanced responsiveness to other specific TLR ligands [131].This discrepancy might be due to differences in the heterogeneity and activation status of target cells [132].

Various TLRs expressed on B cells are not only capable of promoting antibody responses in the absence of T cells help [136, 137], but also promote germinal center (GC) responses very strongly [137–140].TLRs promote GC formation in an antigen oligomerization degree dependent manner [141](82). For example, viral particles with high oligomerized antigen proteins stimulate GC formation using TLR7 or TLR9, this response could be speculated as an adaptive mechanism to promote the generation of protective antibodies against viral infections [141].The TLR7 intrinsic expression is required for effective GC response to generate neutralizing antibodies against Friend virus as retrovirus [142] or LCMV clone 13 [143, 144].Therefore TLRs ability to promote GC formation evolved as a protective mechanism against viral infections however this could also promote lupus-like autoantibodies in a genetically susceptible background [140].

## Conclusion

SLE is a consequence of genetic variants that promote a loss-of-tolerance and systemic autoimmunity against nuclear autoantigens. Half of the SLE patients suffer from some sort of renal involvement. Endogenous nucleic acids do not only serve as autoantigens but also as auto-adjuvants in the pathogenesis of lupus and lupus nephritis because they drive the activation of antigen-presenting cells, and type I interferon release, and consequently adaptive immunity and immune complex disease, e.g. lupus nephritis. Targeting these mechanisms is possible in several ways: 1) avoiding cell death, e.g. sunburns by applying sunscreens, 2) avoiding drugs that endorse hypomethylation of endogenous nucleic acids, e.g. dihydralazine, 3) scavenging nucleic acid debris [145], 4) blocking TLR activation with chloroquine or more specific endosomal TLR antagonists, 5) blocking co-stimulation or 6) blocking type I interferon signaling [146, 147]. Other endogenous triggers of innate immunity activate TLRs on also renal parenchymal cells, e.g. TLR3 in mesangial cells or TLR2 and TLR4 on endothelial cells and podocytes at the glomerular filtration barrier [148]. Altogether, TLRs elicit major contributions to the pathogenesis of SLE and lupus nephritis and their associated pathways remain attractive targets for therapeutic interventions.

## Abbreviations

AP1: Activator protein 1
APRIL: A proliferation-inducing ligand
BLyS: B lymphocyte stimulator
DC: Dendritic cells
dsDNA: Double stranded deoxyribonucleic acid
IC: Immune complex
IFN: Type I interferon
IKK: IκB kinase (αβγε-apha,beta,gamma, epsilon)
IRAKs: IL-1R-associated kinases
IRFs: Interferon-regulatory factors
LN: Lupus nephritis
MYD88: Myeloid differentiation primary-response protein 88
RIP: Receptor-interacting protein kinases
SLE: Systemic lupus erythematosus
ssRNA: Single stranded ribonucleic acid
TAK1: Transforming growth factor beta-activated kinase 1
TIR domain-containing adaptor protein: Tirap toll-interleukin 1 receptor (TIR) domain-containing adaptor protein
TLRs: Toll like receptors
TNF-α: Tumor necrosis factor-α
TRAFs: TNF receptor-associated factors
TRIF: Related adaptor molecule TRAM
